# Brentuximab vedotin: targeting CD30 as standard in CTCL

**DOI:** 10.18632/oncotarget.24472

**Published:** 2018-02-10

**Authors:** H. Miles Prince, Ashish Gautam, Youn H. Kim

**Affiliations:** H. Miles Prince: Division of Cancer Medicine, Peter MacCallum Cancer Centre and Sir Peter MacCallum Department of Oncology and Epworth Healthcare, The University of Melbourne, Victoria, Australia

**Keywords:** brentuximab vedotin, CTCL, CD30, ALCANZA

The recently reported ALCANZA study was the first phase 3 trial to compare a novel systemic agent against standard therapy in cutaneous T-cell lymphoma (CTCL). ALCANZA assessed the anti-CD30 antibody-drug conjugate brentuximab vedotin *vs* physician’s choice of methotrexate or bexarotene in previously treated CTCL, specifically mycosis fungoides (MF) and primary cutaneous anaplastic large-cell lymphoma (pcALCL), expressing the cell surface antigen CD30 [1]. In ALCANZA, unprecedented high rates of clinically meaningful, durable responses were achieved with brentuximab vedotin, which has the potential to change treatment practice in relapsed/refractory (R/R) CTCL.

Currently available systemic therapies for CTCL offer limited efficacy benefits, with low response rates and durations of response [2]. For advanced-stage CTCL, no curative therapies exist, and there is no consensus on the most appropriate systemic therapy. Consequently, unmet need in CTCL remains high, particularly in R/R or advanced-stage disease. The substantial efficacy demonstrated in ALCANZA suggests that brentuximab vedotin could meet this need, particularly since the comparator arm reflected realistic clinical practice, with methotrexate and bexarotene representing the most frequently used treatment options in the setting [3].

Objective response rates (ORRs) with currently available systemic CTCL therapies range from 30-40% [4]. However, ORR is not necessarily the most valuable measure of efficacy: criteria for response assessment are not standardized across studies, and ORR does not capture response durability, an important aspect of effective treatment [5]. ALCANZA assessed a novel primary endpoint: the proportion of patients achieving an objective global response (comprising measurements in skin, node, viscera, and blood) lasting at least 4 months (ORR4) [1]. ORR4 provides both rate and duration of response, and is not confounded by subsequent therapies, so represents a clinically meaningful endpoint for CTCL. At a median follow-up of 22.9 months, a significantly superior ORR4 was observed in ALCANZA patients treated with brentuximab vedotin compared with physician’s choice (*n* = 64 in each arm; ORR4 56.3% *vs* 12.5%; *p* < 0.0001). The proportion of patients achieving an objective response of any duration was also higher with brentuximab vedotin *vs* physician’s choice (67% *vs* 20%; *p* < 0.0001), as was the complete response rate (16% *vs* 2%; *p* = 0.0046). Additionally, a significant progression-free survival benefit was shown with brentuximab vedotin over physician’s choice (median 16.7 *vs* 3.5 months; hazard ratio (HR) 0.27 (95% confidence interval 0.17-0.43); *p* < 0.0001), and time to next therapy was 14.2 *vs* 6.1 months; HR 0.335; *p* < 0.001 [1].

ORR4 was high with brentuximab vedotin across key ALCANZA subgroups, including CTCL subtypes (pcALCL and MF) and in skin-only or extracutaneous disease. Alongside MF and pcALCL, brentuximab vedotin has previously demonstrated activity in patients with Sézary syndrome and lymphomatoid papulosis [6, 7], who were excluded from ALCANZA; this represents an attractive area for future research.

In ALCANZA, CD30 positivity was defined as ≥10% CD30-positive malignant cells or lymphoid infiltrate in ≥1 biopsy samples. There was no correlation between CD30 expression and ORR4, with sustained responses occurring across the range of CD30 expression levels [1-8]. Since brentuximab vedotin is also active in lesions with low CD30 expression [6, 7], this highlights the complexity of the relationship between CD30 expression and response to CD30-targeted therapy. Techniques for measurement of CD30 expression vary in sensitivity, reliability, and reproducibility, and there is no consensus on the definition of CD30 positivity. To further understand the role of CD30 as a treatment target in CTCL, it is essential that measurement techniques and interpretation are standardized in future.

Advanced-stage CTCL is associated with reduced patient quality of life (QoL). Symptoms including pain, pruritus, and fatigue place a significant physical burden on patients, and the chronic and morbid nature of the disease is detrimental to psychological and emotional wellbeing. Brentuximab vedotin-treated patients in ALCANZA experienced meaningful reduction in patient-reported skin symptom burden, using the Skindex-29 measure, showing significant reduction in symptoms with brentuximab vedotin *vs* physician’s choice [1].

Adverse events (AEs) are also associated with impaired QoL, and AE profiles for individual agents may vary between diseases. However, AEs reported in ALCANZA were consistent with the established tolerable and manageable safety profile of brentuximab vedotin [1]. While the incidence of grade 3/4 AEs was similar in both treatment arms, the overall AE frequency and rate of AE-related treatment discontinuation was higher with brentuximab vedotin, but this is perhaps unsurprising given the substantially longer treatment exposure compared with methotrexate or bexarotene. The majority of discontinuations were attributable to peripheral neuropathy (PN), a known toxicity of brentuximab vedotin. PN was reported in 67% of brentuximab vedotin-treated patients, but led to treatment discontinuation in only 14%; it was generally manageable and reversible with dose adjustment.

The results of the ALCANZA study provide compelling evidence that brentuximab vedotin is now an important treatment option for patients with R/R CD30-positive pcALCL and MF. Indeed, in November 2017, the Food and Drug Administration approved brentuximab vedotin for use in this setting in the USA, and European Medicines Agency recommendation for approval was received in December 2017. Moreover, looking forward, brentuximab vedotin may offer promise in other settings, including in patients with lesions exhibiting low CD30 expression, in other CD30-positive CTCL subtypes, or as retreatment in CTCL, providing a potential post-relapse option. Different dosing schedules are also of interest; dose reduction is a key tactic in AE management, and comparative studies could assess the impact of altered dosing schedules on safety and efficacy. Brentuximab vedotin may offer potential in combination with other therapies; combination regimens could provide another step forward in identifying treatments that further improve and prolong responses.

## References

[1] Prince HM (2017). Lancet.

[2] Hughes CF (2015). Blood.

[3] Quaglino P (2017). Ann Oncol.

[4] Scarisbrick JJ (2016). Curr Opin Oncol.

[5] Olsen EA (2011). J Clin Oncol.

[6] Duvic M (2015). J Clin Oncol.

[7] Kim YH (2015). J Clin Oncol.

[8] Kim YH (2017). J Clin Oncol.

